# Eco-friendly and solar light-active Ti-Fe_2_O_3_ ellipsoidal capsules’ nanostructure for removal of herbicides and organic dyes

**DOI:** 10.1007/s11356-022-23119-0

**Published:** 2022-10-06

**Authors:** Hanan H. Mohamed, Dina H. A. Besisa

**Affiliations:** 1 Chemistry Department, Faculty of Science, Helwan University, Ain Helwan, Cairo, 11795 Egypt; 2 Refractory & Ceramic Materials Division (RCMD), Central Metallurgical R&D Institute (CMRDI), Helwan, P.O. Box 87, Cairo, 11421 Egypt

**Keywords:** Green Ti-Fe_2_O_3_, Ellipsoidal capsules, Solar photocatalysis, BPB dye, 2,4-D

## Abstract

In this work, Ti-doped Fe_2_O_3_ with hollow ellipsoidal capsules nanostructure has been prepared in a green manner using plant extract (flax seed). This new green hematite nanomaterial has been evaluated as photocatalyst for water treatment by testing its activity for degradation of bromophenol blue dye (BPB) and 2,4-dichlorophenoxy acetic acid (2,4-D) herbicide. For a better understanding of the green material properties, a comparison with the pristine Fe_2_O_3_ nanospheres previously prepared by the same procedure is included. Structural and optical properties of the green prepared materials are studied. The results revealed the success doping of Ti^4+^ at Fe^3+^ site, without forming any of TiO_2_ phases. It was also found that the Ti doping resulted in the reduction of the band gap of Fe_2_O_3_ as well as changing the morphology. The Ti-doped Fe_2_O_3_ nanomaterial exhibited an enhanced photocatalytic activity either for BPB dye or for 2,4-D degradation with more than 2 times higher rate than that using pristine Fe_2_O_3_.

**Supplementary Information:**

The online version contains supplementary material available at 10.1007/s11356-022-23119-0.

## Introduction

The world is facing a critical shortage of clean water supplies as resulted from global population growth and industrialization. Most of our water resources including surface and ground water are so polluted and dangerous that it cannot even be used for industrial purposes. Dyes exist in water from textile, leather, and tuning industries represent a growing environmental concern (Sharma and Bhattacharya 2017; Liu 2020). Dye molecules possess aromatic rings in their structure, which render them with a high biodegradation resistance, high toxicity, and carcinogenic. In addition, they prevent the penetration of solar light and delay the photosynthetic reaction which significantly affects the aquatic life (Lellis et al. 2019). Moreover, about 80% of pesticides and herbicides are directly leached into groundwater causing major environmental issue. Besides being carcinogenic, pesticides and herbicides are regarded as endocrine disrupting chemicals, causing adverse effects on the endocrine system, reproductive system, and immunologic system of human and animals (Syafrudin et al. 2021). Photocatalysis using semiconductor nanomaterials and solar energy is considered as the most promising solution to address the challenges concerning water purification from such organic, inorganic, and biological pollutants (Ren et al. 2021, Al Qarni et al. 2019; Mohamed and Alsanea 2020; Mohamed et al. 2012). However, the photocatalytic efficiency of semiconductor nanomaterials is still limited by low activity and weak solar light harvesting that makes the photocatalytic system not applicable in real (Besisa and Ewais 2019; Besisa et al. 2016). Tremendous efforts have been devoted to address this issue tailoring photocatalytic nanomaterials with different types and microstructures (Thongam and Chaturvedi 2021).

Among metal oxide semiconductors, hematite (Fe_2_O_3_) has drawn scientific interest due to its outstanding properties such as chemical and thermodynamical stability, high solar light absorptivity (absorbs ~ 40% of visible light), and non-toxicity (Asif et al. 2021; Mishra and Chun 2015). Various physical and chemical methods have been reported on the synthesis of hematite nanomaterials such as co-precipitation (Fouad et al. 2019), thermal decomposition (Samrot et al. 2021), sol–gel (Samrot et al. 2021), and hydrothermal method (Tadic et al. 2019). Nowadays, green synthetic method of hematite using plant extract has attracted the scientific community as being clean, cheap, simple, and safe, in addition to their enhancement of the nanoparticle’s morphology (Mohamed et al. 2019; Al-Hakkani et al. 2021; Rostamizadeh et al. 2020).

Despite the characteristic properties of Fe_2_O_3_, its small band gap (1.9–2.2 eV) reduces its catalytic performance due to low conductivity and rapid recombination of charge carriers (Li and Chu 2018). Several attempts were applied to overcome this problem such as doping (Yina et al 2018), modifying nanostructure (Chen and Lin 2014), or coupling with other semiconductor (Bora 2016). An effective process is doping with other transition metal ions, such as Zn^2+^ (Suman et al. 2020), Ni^2+^ (Liu et al. 2012), Co^2+^ (Keerthana et al 2021), Al^3+^ (Kleiman-Shwarsctein et al. 2010), Sn^4+^ (Popov et al. 2022; Em et al. 2022), and Ti^4+^ (Fu et al 2014; Biswas et al. 2020). Generally, during doping, the orbital hybridization takes place between the dopant orbital and molecular orbital of host, which leads to a tunable electronic structure and controllable potentials of the VB and the CB. Doping by transition metal ions leads to the generation of new energy levels within the bandgap area (donor level above the VB or acceptor level below the CB) of the photocatalyst. This results in sub-band-gap irradiation from which the electrons can be excited from the d-band of the dopant to the CB of the host photocatalyst or from the VB of the host photocatalyst to the d-band of the dopant by photons with lower energy than that required by the un-doped photocatalyst (Shao et al. 2018). Moreover, the importance of transition metal doping is represented by the formation of the trapping levels and their ability to tune some properties of the semiconductors such as electrical, optical, and therefore photocatalytic properties. For instance, the non-isovalent substitution of Zn^2+^ at Fe^3+^ site resulted in charge imbalance in Fe_2_O_3_ lattice. Three mechanisms have been proposed to preserve the neutrality of charges, including Fe^3+^  → Fe^2+^ transformation, creation of cation vacancies, and filling of oxygen vacancies (Suman et al. 2020). In addition, doping with tetravalent metal ion, which can form a covalent bond with the oxygen, leads to increase the number of charge carriers, hence increasing the conductivity. For example, doping of with Sn^4+^ ions has greatly improved gas sensing and photoelectrochemical properties of Fe_2_O_3_ nanoparticles and thin films (Popov et al. 2022). Similarly, Ti-doped Fe_2_O_3_ can greatly enhance the electron–hole pair separation as well as increase the charge density, therefore improves the photocatalytic and photoelectrochemical activity (Fu et al 2014; Biswas et al. 2020). It was hypothesized that the enhanced performance of Ti-treated hematite is due to the formation of Fe_2_TiO_5_-instead of substitution of Fe in Fe_2_O_3_ by Ti (Deng et al. 2015).

Several studies have been reported on producing Ti-treated (or doped) hematite nanostructure arrays for solar water splitting and electrochemical applications (Fu et al. 2014; Biswas et al. 2020; Deng et al. 2015). Rtimi and co-workers have extensively studied Ti/Fe oxides and the effect of different Fe:Ti ratio on the morphological optical properties and on the photocatalytic and antibacterial activity under solar light irradiation (Nardi et al. 2015; Rtimi et al. 2015, 2016). To the best of our knowledge, none of the previous studies on Ti and Fe oxides paid attention to green production of Ti-doped hematite nanostructures or using the produced Ti-treated Fe_2_O_3_ nanomaterials for photocatalytic degradation of organic pollutants. Therefore, our work focuses on eco-friendly production of Ti-doped Fe_2_O_3_ nanostructure, using plant extract, and studied their structural, optical, and morphological properties in comparison with pristine Fe_2_O_3_. Furthermore, the effect of Ti doping on the photocatalytic degradation of organic pollutants has been investigated and the mechanism proposed.

## Experimental

### Materials synthesis

Flax seed extract was prepared according to previous work (Mohamed et al. 2019). Typically, a specific amount of washed and grinded FS powder was dissolved in distilled water then heated until boiling. The resulted suspension of FS extract was filtered and then stored in the fridge for further use.

Ti-Fe_2_O_3_ nanoparticles were synthesized from Fe(NO_3_)_3_.9H_2_O as α-Fe_2_O_3_ precursor and TTIP (Ti{OCH(CH_3_)_2_}_4_) as Ti source. Typically, FSE suspension is drop-wise added to an aqueous Fe^3+^ solution under ultrasonic vibration for 2 h at 50 °C. After that, 5% of TTIP is dropwise added to the above suspension and left stirring for 30 min. The final solid products were separated from the formed suspension by centrifugation, washed two times by distilled water using centrifugation, and then dried at 70 °C for 1 h. Pure Fe_2_O_3_ was prepared by the same method without adding TTIP.

### Characterization

XRD measurements were performed using a Cu-K x-ray with tube conditions of 40 kV. SEM images were taken by using FEI, Inspect S50 at accelerating voltage of 20 kV. TEM analysis was performed on TEM FEI, Morgagni 268, Brno, Czech Republic. DRS measurements were recorded on Jasco V760 spectrometer and UV–vis absorption measurements were recorded on UVD-3200, LABOMED.

### Photocatalytic activity

The photocatalytic activity of hematite nanomaterials was evaluated for the degradation Bromophenol Blue (BPB) dye as model dye and 2,4-dichlorophenoxyacetic acid (2,4-D) as model herbicide. In a typical photocatalytic experiment, 1 g/L of the photocatalyst was ultrasonically dispersed in pure water. Then, 20 mg/L aqueous solution of the water pollutant (BPB dye or 2,4-D) was added to the catalyst’s suspension. Prior to the photoirradiation, the adsorption of the organic molecules on the surface of the photocatalyst was tested by stirring the photocatalyst/organic pollutants (BPB or 2,4-D) suspension in the dark for 30 min. After that, the photo-irradiation was carried out at room temperature using a solar lamp (30 W) under stirring. The photocatalytic experiments have been repeated 3 times. To determine the change in the concentration of the organic pollutant with time, liquid samples of the photocatalyst/pollutant suspension were taken, filtered from the photocatalyst particles, and then measured by the UV–vis spectrophotometer.

The photocatalytic degradation efficiency has been determined using the following equation:

$$\mathrm{Photodegradation}\;\mathrm{Efficiency}\;\%=\frac{C_0-C_t}{C_0}\times100=\frac{A_0-A_t}{A_0}\times100$$

where C_0_ is the initial concentration of pollutant and C_t_ is the pollutant’s concentration at certain reaction time t (min). The kinetics of the photocatalytic experiments has been determined by plotting $-ln\frac{{C}_{t}}{{C}_{0}}$ versus time which should yield straight lines slope of the apparent first-order rate constant *k* according to the following equation:

$$-ln\frac{{C}_{t}}{{C}_{0}}=kt$$

## Results and discussion

### Materials characterization

#### Characterization of FSE

UV–vis DRS of FSE shows a broad absorption in the visible region which is attributed to the presence of antioxidants in the FSE (see Figure S1(a)). FTIR spectroscopic measurement of FSE (Figure S1(b)) confirms the presence of polyphenolic and phenolic compounds as indicated from the band at ~ 3400 cm^−1^ for -OH stretching vibration (Butsat and Siriamornpun 2010). The GC–MS measurement confirms the existence of carbohydrate, esters, and cyclononasiloxane compounds in addition to polyphenolic compounds (Figure S1(c)) (Mohamed et al. 2019). Based on this, the compounds in the aqueous extract of FS are supposed to act as inducing and stabilizing agent during the formation of the nanomaterials. The mechanism of preparation of Ti-Fe_2_O_3_ nanomaterial using FSE can be described by the following equation:

1 $${2Fe}^{3+}+4OH-\left[FSE\right]\stackrel{Ultrasound, 50^\circ C}{\to }2FeOOH+ {2H}^{+}$$

2 $$2FeOOH+{Ti}^{4+} \stackrel{500^\circ C}{\to }{Ti-Fe}_{2}{O}_{3}+{H}_{2}O$$

#### Characterization of nanomaterials

**Fig. 1:**
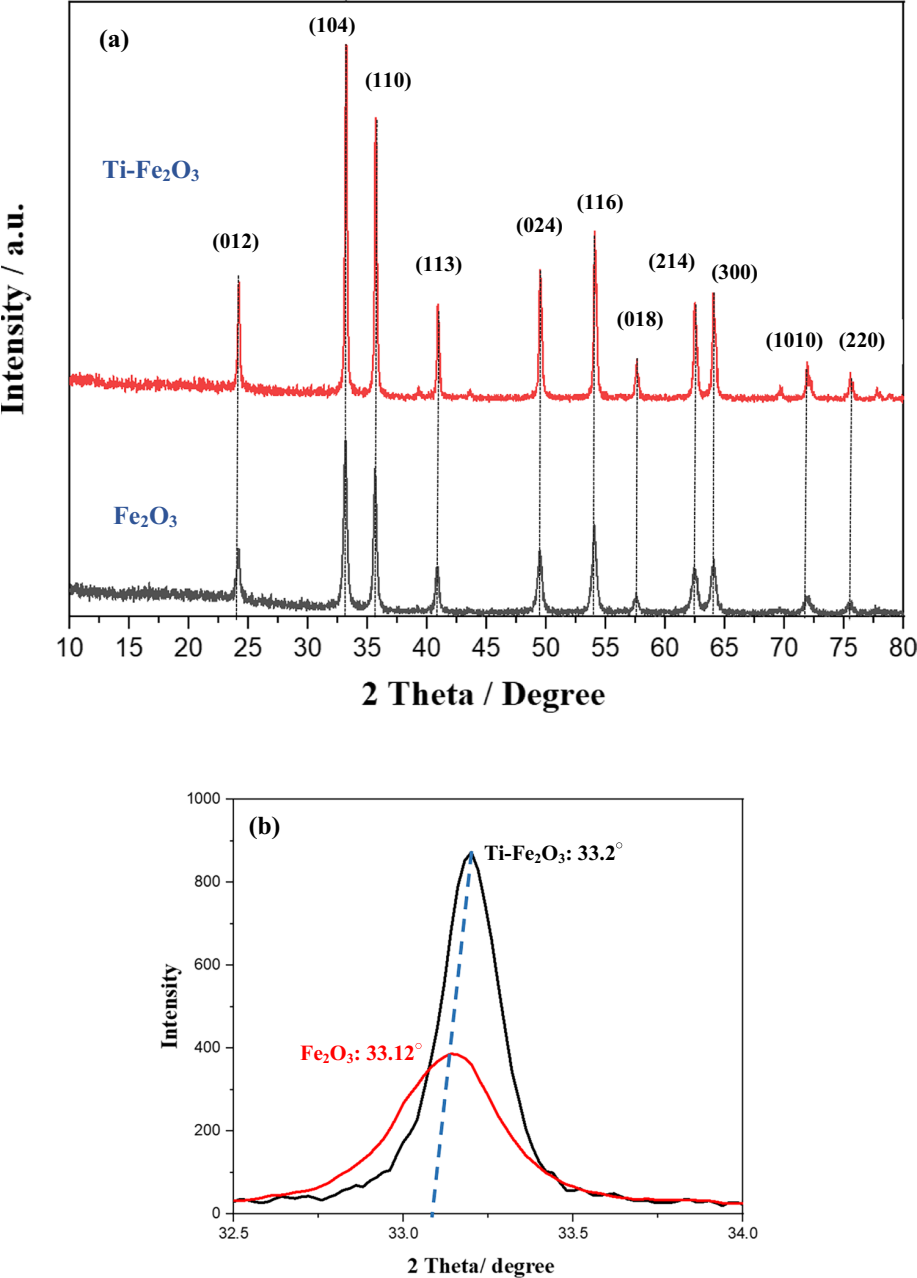
**a** XRD patterns of Fe_2_O_3_ and Ti-Fe_2_O_3_ nanomaterials and **b** enlargement of the scale to show the shift of the diffraction angels between pure Fe_2_O_3_ and Ti-Fe_2_O_3_

The XRD patterns of hematite nanomaterials are shown in Fig. 1a. The diffraction peaks of both pure Fe_2_O_3_ and Ti-Fe_2_O_3_ are well indexed to rhombohedral hematite at 24.13°, 33.15°, 35.612°, 40.85°, 49.48°, 54.09°, 57.59°, 62.41°, and 63.99°. The XRD results revealed the formation of pure and well crystalline α-Fe_2_O_3,_ which are in good agreement with the previous reports (Mohamed et al. 2019; Rahman and Joo 2013). The identical XRD patterns of pure- and Ti doped Fe_2_O_3_ indicating that Ti^4+^ ions have substituted, at least partially, Fe^3+^ ions in the hematite matrix without changing the rhombohedral structure. Moreover, the intensity of the peaks is higher for Ti-Fe_2_O_3,_ and its half height width is also higher than pure hematite. The results revealed the increase of the grain size of the Ti doped hematite. In addition, small shift to a larger diffraction angle is obvious for the diffraction peaks of Ti-Fe_2_O_3_ (see Fig. 1b). This shift has been correlated to the substitution of the smaller Ti^4+^ ions (ionic radius = 0.061 nm) into the larger Fe^3+^ ions (ionic radius = 0.069 nm) of Fe_2_O_3_ (Hwang and Jung 2022) (Fig. 1b).

**Fig. 2:**
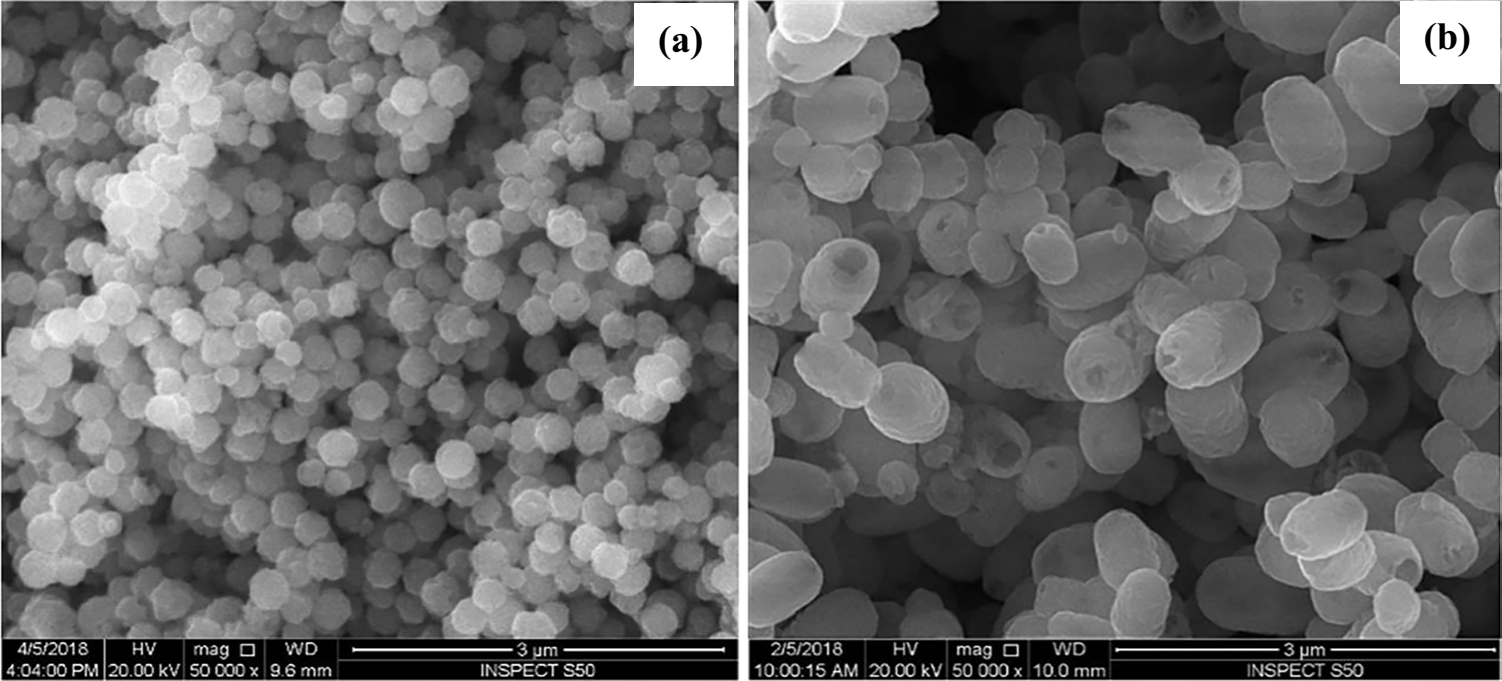
SEM images of Fe_2_O_3_ (**a**) and Ti-Fe_2_O_3_ (**b**) nanomaterials

**Fig. 3:**
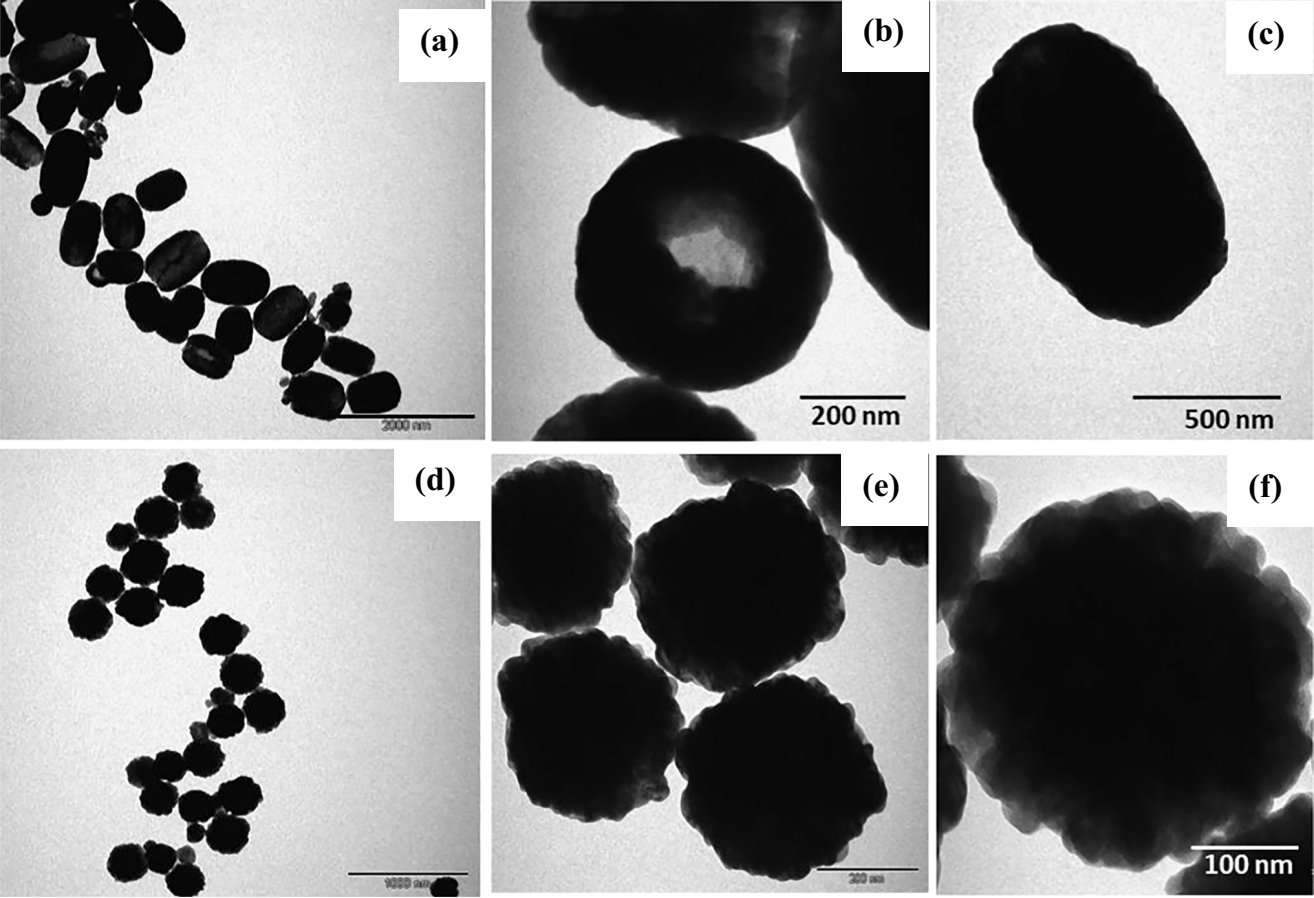
TEM images of Fe_2_O_3_ (**a**, **b**, **c**) and Ti-Fe_2_O_3_ (**d**, **e**, **f**) nanomaterials

The morphological features of the pure and Ti doped Fe_2_O_3_ were investigated by SEM and TEM. SEM image (Fig. 2) shows the homogeneous spherical shaped particles of α-Fe_2_O_3_, while the SEM image of Ti-Fe_2_O_3_ shows homogeneous open hollow ellipsoidal capsules structure. TEM images of pure and Ti-doped Fe_2_O_3_ nanostructures are shown in Fig. 3. Nanospheres of pure Fe_2_O_3_ have diameter range of 100–300 nm, while ellipsoidal capsules of Ti-doped Fe_2_O_3_ have width range of 400–500 nm and length range of 600–800 nm. The change in the morphology of hematite upon Ti doping can be attributed to the synthesis procedure applied in this work since the in situ methods usually alter the crystalline structure and morphology of the doped material, in addition to the effect of the dopant precursor during nanoparticle growth (Kusior et al. 2019).

**Fig. 4:**
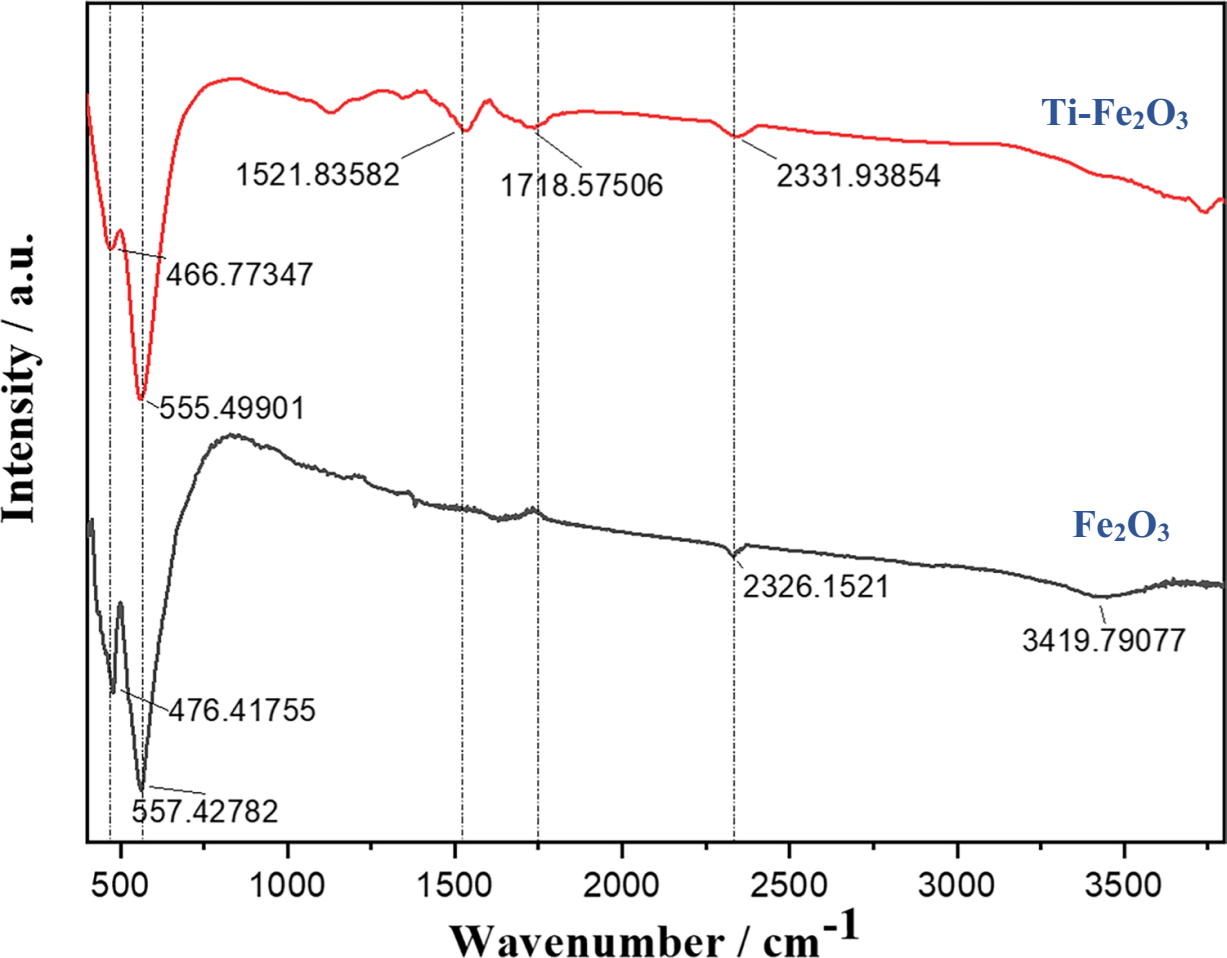
FTIR spectra of Fe_2_O_3_ and Ti-Fe_2_O_3_ nanomaterials

The FTIR spectra of pure and Ti-Fe_2_O_3_ nanostructures show two main peaks at 467–476 cm^−1^ assigned to bending vibration of O–Fe–O and at 555–557 cm^−1^ referred to the stretching vibrations of Fe–O bonds (Mohamed et al. 2019; Rahman and Joo 2013; Zielinska et al. 2010). The peaks at 1625 and ~ 3420 cm^−1^ are corresponding to the –OH stretching vibrations (Fig. 4) (Mohamed et al. 2019; Rahman and Joo 2013; Zielinska et al. 2010). By a comparison with pure Fe_2_O_3_, the broad peak at 3420 cm^−1^ is suppressed for Ti-Fe_2_O_3_ which may be due to a lower hydroxylation level due to Ti doping. It is concluded that the non-isovalent substitution of Ti^4+^ at Fe^3+^ sites can induce structural modifications of the hematite surface, preventing from reaching the full hydroxylation.

**Fig. 5:**
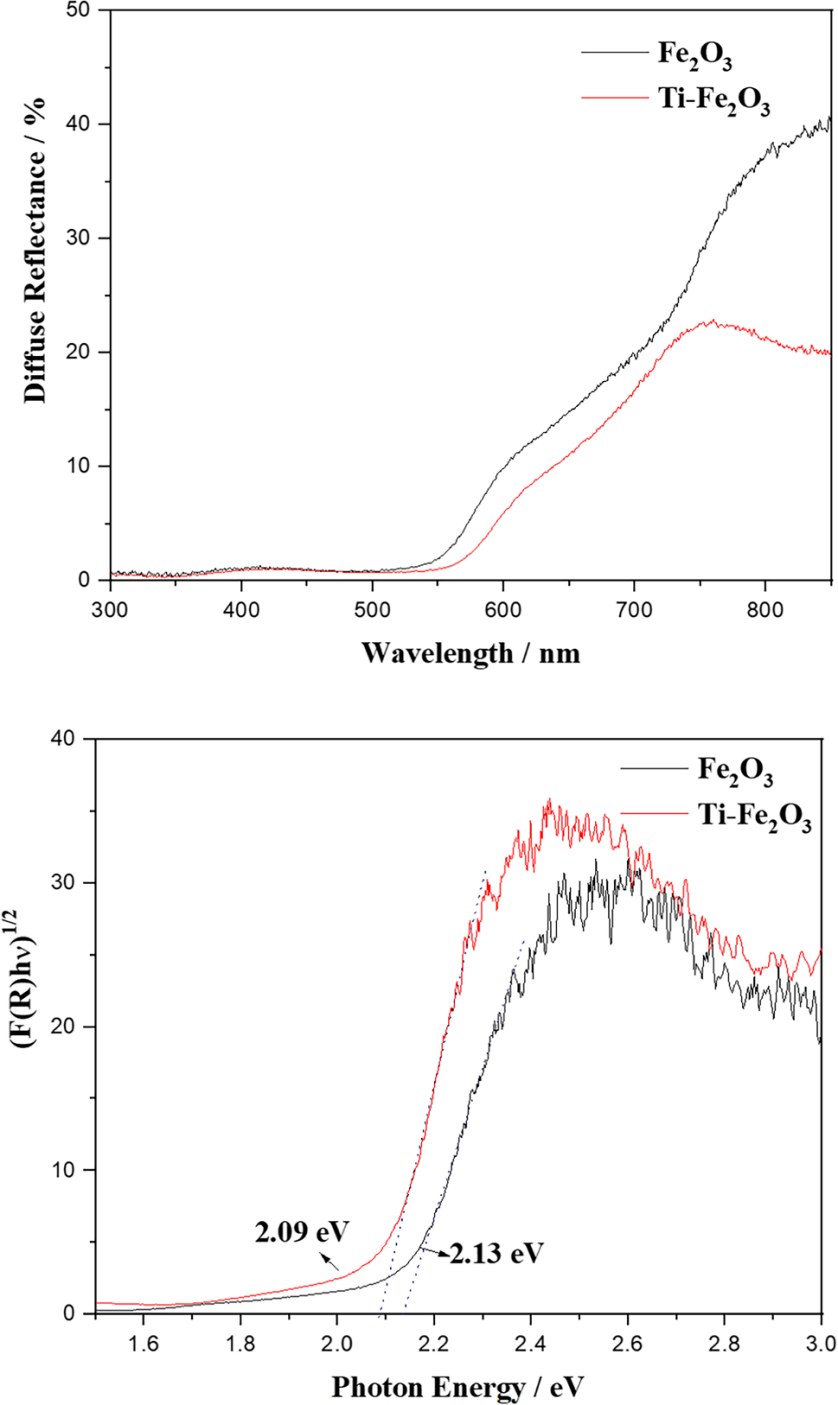
UV–vis diffuse reflectance (**a**) and Kubelka–Munk plot (**b**) of Fe_2_O_3_ and Ti-Fe_2_O_3_ nanomaterials

UV–vis diffuse reflectance measurements (Fig. 5a) show high absorption in the visible region with absorption edge of ~ 580 nm for pure Fe_2_O_3_. The absorption edge is shifted to ~ 610 nm for Ti-Fe_2_O_3_. The band gap energy of the samples has been estimated from the intercept of the tangents of Kubelka–Munk plots to be 2.13 and 2.03, for pure Fe_2_O_3_ and Ti-Fe_2_O_3_, respectively (Fig. 5b). The shift of the band gap energy of Ti-doped Fe_2_O_3_ to lower energy can be attributed to the increase in structural disorder or defects with Ti doping. This decrease in the band gap of Ti-Fe_2_O_3_ compared with the Fe_2_O_3_ could be also attributed to the introduction of additional energy level below the conduction band of Fe_2_O_3_ by Ti doping.

### Photocatalytic activity

#### Photocatalytic degradation of BPB

**Fig. 6:**
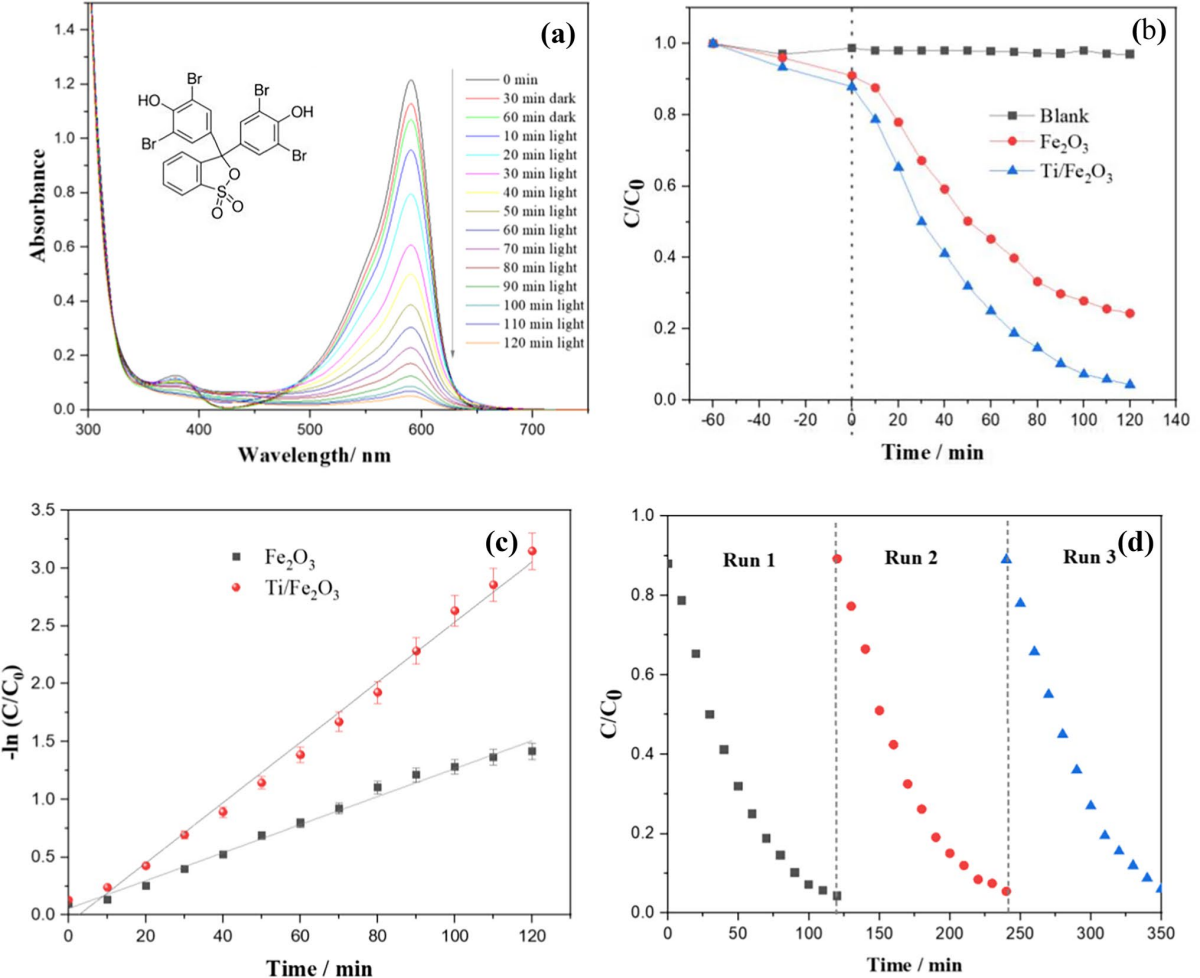
**a** UV–vis absorption spectra of an aqueous solution of BPB during solar light illumination using Ti-Fe_2_O_3_, **b** efficiency of the photocatalytic degradation of BPB as the variation of C/C_0_ with irradiation time, **c** linear plots of − ln C/C_0_ vs time for the experimental data in **b**, and **d** recyclability of Ti-Fe_2_O_3_

Figure 6a shows the absorption spectra for degradation of BPB dye solution in the presence of Ti-Fe_2_O_3_ under solar light irradiation. The UV–vis absorption spectrum of BPB shows a maximum absorbance at 590 nm and another small peak at 380 nm. The absorption peak at 590 nm decreased rapidly with irradiation time. After 2 h of light irradiation, about 95% of the dye has been degraded. For comparison, BPB degradation has been evaluated using pure Fe_2_O_3_ at the same experimental conditions. The photocatalytic degradation mechanism of BPB has been studied in detail using GC–Ms (Mohamed and Youssef 2017). It was shown that ^•^O_2_^−^ and ^•^OH radicals attack the dye molecules leading to ring cleavage to form hydroquinone, bromophenol, and pentenethiol intermediates, which will be finally mineralized forming CO_2_, H_2_O, and SO_4_^2−^. The degradation efficiency of both nanomaterials is shown in Fig. 6b. The pseudo-first order kinetics of the absorption data is shown in Fig. 6c. The observed rate constant (k_obs_) is estimated as 0.012 and 0.026 min^−1^ for pure Fe_2_O_3_ and Ti-Fe_2_O_3_, respectively, indicating that the photocatalytic activity of the Ti-Fe_2_O_3_ is more than twofold higher than that of pure Fe_2_O_3_ nanomaterial. The results can be explained by the improvement of conductivity and photoactivity of hematite due to the non-isovalent substitutional doping by introducing Ti^4+^ into hematite. Though there is an argument on explaining the improvement of photoactivity of Ti-Fe_2_O_3_ on whether the effect of Ti^4+^ ions as donor, or due to the small polaron hopping (Fe^3+^ + Ti^4+^ → Fe^2+^ + Ti^3+^), it has not been proved in our study. Probably, the photogenerated electrons could be trapped at the surface Ti^4+^ sites forming Ti^3+^, promoting the electron–hole pair separation, and enhancing the photocatalytic activity. The trapped electrons at Ti^3+^ can be then react to the adsorbed O_2_ forming reactive ^•^O_2_^−^.

The recyclability of Ti-Fe_2_O_3_ was tested for 3 runs used in degradation of BPB (Fig. 6d). No noticed decrease in the activity of the photocatalyst was observed revealing the high stability of the Ti-Fe_2_O_3_ nanomaterial.

#### Photocatalytic degradation of 2,4-D

**Fig. 7:**
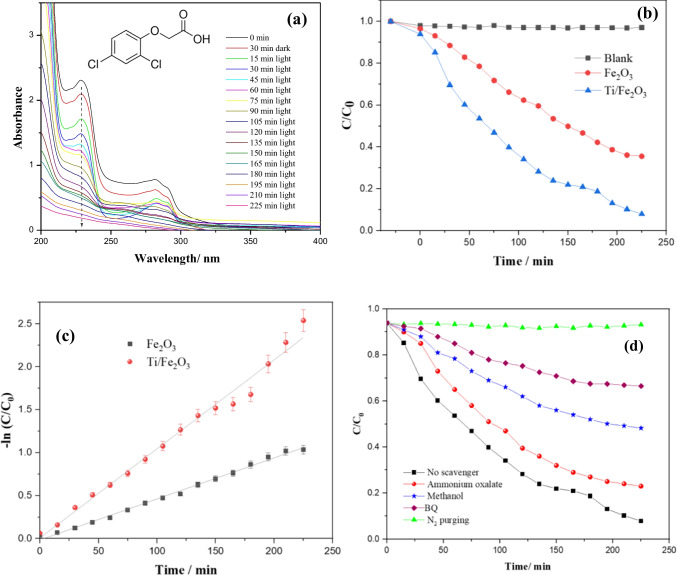
**a** UV–vis absorption spectra of an aqueous solution of 2,4-D during solar light illumination using Ti-Fe_2_O_3_, **b** efficiency of the photocatalytic degradation of 2,4-D as the variation of C/C_0_ with irradiation time, **c** linear plots of − ln C/C_0_ vs time for the experimental data in **b**, and **d** photocatalytic degradation of 2,4-D in the presence of different scavengers for h + , OH^•^, and O_2_^•−^

The photocatalytic degradation of 2,4-D, an organic pollutant frequently exists as the agricultural effluent, was also evaluated using hematite nanomaterials. Figure 7a shows the UV–vis absorption spectra for the degradation of 2,4-D under solar light irradiation in the presence of Ti-Fe_2_O_3_. The UV–vis spectrum of 2,4-D shows 2 main peaks at 285 and 230 nm. The peak at 285 nm is specified to the n → π* transitions of the C–Cl bonds and the peak at 230 nm is assigned to π → π* transition of aromatics rings (González et al. 2020). After 30 min in the dark, ~ 6% of the herbicide molecules have been adsorbed on the surface of Ti-Fe_2_O_3_. After solar light irradiation, the characteristic peak at 230 nm was found to be decreased rapidly with irradiation time, while, from 30 to 90 min irradiation, a significant increase in absorbance is observed in the region 240–270 nm which can be attributed to the formation of benzoquinones intermediates [35]. Moreover, higher decrease in the intensity is observed for the peak at 230 nm as compared to the peak at 285 nm, indicating that the C = C bonds in the aromatic ring of 2,4-D molecule are more likely to be degraded by ^•^O_2_^−^ and ^•^OH than the C–Cl bond. After ~ 4 h, almost all 2,4-D has been degraded. Thus, 2,4-D is mainly degraded by electrophilic attack of hydroxyl radical to aromatic compounds and C–Cl bands, to produce primary organic acid such as acetic and formic acid (Ng et al. 2010), which are finally mineralized to CO_2_ and H_2_O. The degradation kinetics of 2,4-D has been evaluated by monitoring the change in the absorption at 230 nm (Fig. 7b). The pseudo-first-order kinetics of the absorption data is shown in Fig. 7c with k_obs_ 0.0048 min^−1^ and 0.0109 min^−1^, for pure Fe_2_O_3_ and Ti-Fe_2_O_3_, respectively. It is obvious that the photocatalytic degradation efficiency of the herbicide compound is more than two times higher using Ti-Fe_2_O_3_ as compared to pure Fe_2_O_3_ nanomaterial. The kinetic data are summarized in Table 1. This enhancement in the photocatalytic activity can be readily explained by the enhancement of the photogenerated charge separation upon Ti doping. It has been also reported that the photogenerated holes can be captured and stored at Ti^4+^ sites on the surface of Fe_2_O_3_, facilitating their transfer to the surface reactant (Liu et al. 2017). In addition, the higher photocatalytic activity can be attributed to the synergistic effect of the high specific area of the open hollow ellipsoidal capsule’s structure of Ti-Fe_2_O_3_ and the enhanced charge separation, upon Ti doping.

**Table 1:** Kinetic parameters for the degradation of BPB and 2,4-D using hematite nanomaterials

Nanomaterial	Reactant	k (min^−1^)	*R*^2^
Fe_2_O_3_	BPB	0.012	0.99
Ti/Fe_2_O_3_	BPB	0.026	0.99
Fe_2_O_3_	2,4-D	0.0048	0.99
Ti/Fe_2_O_3_	2,4-D	0.0109	0.98

To identify the reactive species in the photocatalytic degradation of 2,4-D using Ti-Fe_2_O_3_ nanomaterial, a series of trapping tests has been performed. One millimolar of ammonium oxalate, benzoquinone (BQ), and methanol has been used as trapping agents for h^+^, ^•^OH, and ^•^O_2_^‒^ radicals, respectively. In addition, the photocatalytic experiment was performed under N_2_ gas purging to study the effect of dissolved O_2_. Figure 7d shows the photocatalytic degradation efficiency of 2,4-D in the presence of the different scavengers. Based on the results, the photocatalytic degradation of 2,4-D was remarkably inhibited by ^•^O_2_^‒^ scavenger (BQ), less affected by ^•^OH scavenger (methanol), while h^+^ scavenger (ammonium oxalate) is found to has the minor role. In addition, the photocatalytic experiment under N_2_ purging demonstrates the predominant role of dissolved oxygen.

**Fig. 8:**
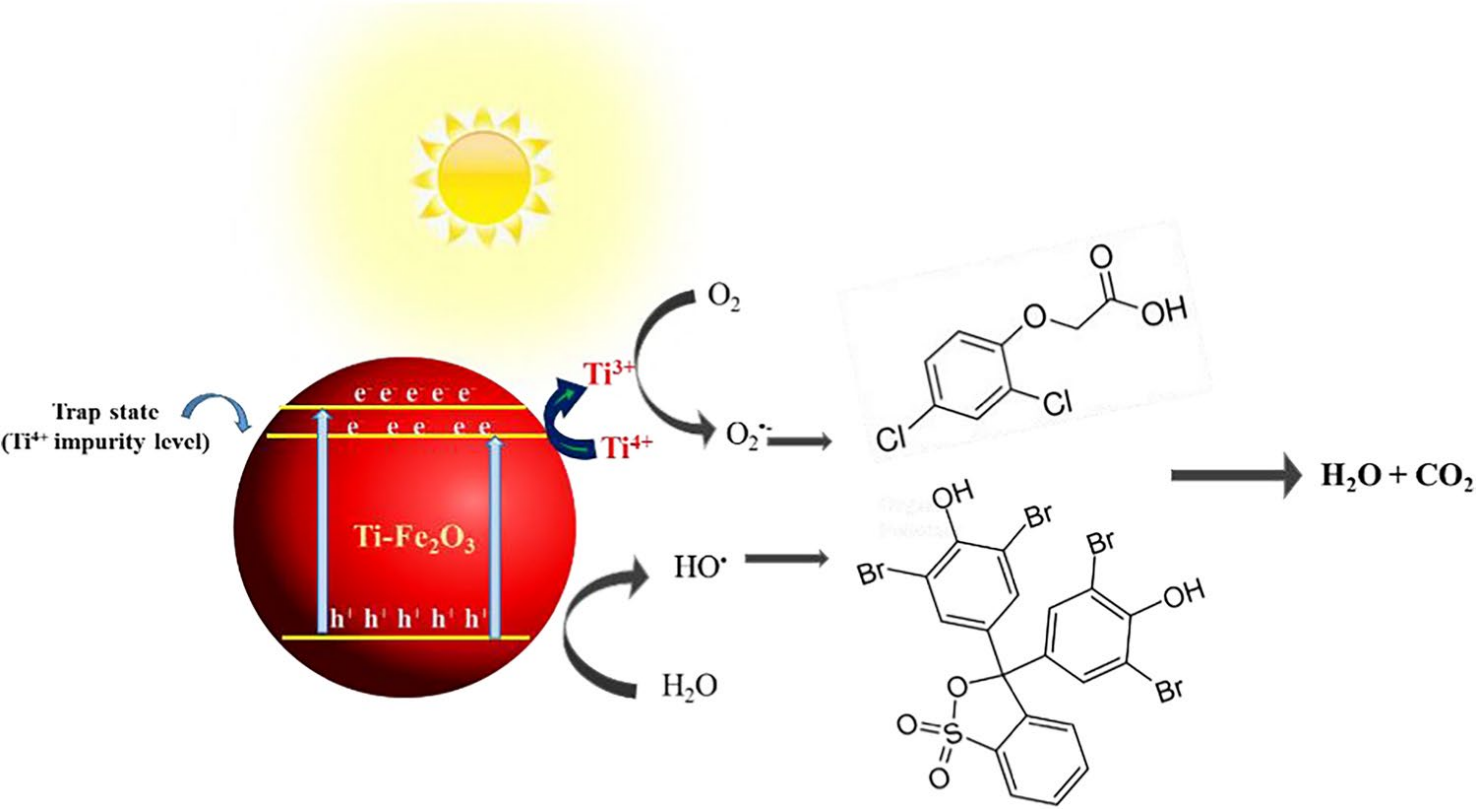
Proposed mechanism for the photocatalytic degradation of BPB dye and 2,4-D using Ti-Fe_2_O_3_ photocatalyst

Based on the above results, and the estimated band gap energy of α-Fe_2_O_3_ (2.13 eV) and of Ti-Fe_2_O_3_ (2.09 eV), the photocatalytic mechanism of Ti-Fe_2_O_3_ nanomaterial can be proposed as follows: the electron–hole pair will be generated under solar light irradiation. The photogenerated electrons on CB of Ti-Fe_2_O_3_ can be trapped at the surface Ti^4+^ sites to give Ti^3+^, enhancing the electron–hole pair separation. While the photogenerated holes reacted to the adsorbed OH^−^/H_2_O forming ^•^OH, the Ti^3+^ species reacted with the adsorbed O_2_ forming ^•^O_2_^−^. Both ^•^O_2_^−^ and ^•^OH radicals will be used for the degradation of organic pollutants (BPB dye or 2,4-D) (Fig. 8).

## Conclusions

In summary, pristine and Ti-doped Fe_2_O_3_ has been successfully prepared in green manner using FS extract. Structural, optical, and morphological properties of the green hematite nanomaterials have been studied by XRD, FTIR, SEM, TEM, and DRS. XRD and FTIR confirmed the success doping of Ti^4+^ at Fe^3+^ site of hematite without forming new phases (e.g., TiO_2_). SEM and TEM showed the change in the morphology of the hematite upon Ti doping, which was attributed to the effect of the in situ doping method as well as the effect of the dopant precursor. DRS results showed an increase in the absorption edge for the Ti-Fe_2_O_3_, and the Kubellka-Munk estimated band gap energy was found to be reduced to be 2.03 eV as compared to pristine Fe_2_O_3_ (2.13 eV). This reduction in the band gap was attributed to the increase in structural disorder or defects with Ti doping as well as to the introducing of additional energy level below the conduction band of Fe_2_O_3_ upon Ti doping. Furthermore, the photocatalytic activity of undoped and Ti-doped hematite nanomaterials was studied for the degradation of BPB dye and 2,4-D herbicides as model water pollutants. The photocatalytic degradation efficiency of BPB dye as well as herbicide compound was found to be more than two times higher using Ti-Fe_2_O_3_ as compared to pure Fe_2_O_3_ nanomaterial. This enhancement of the activity was readily due to the enhancement of e^−^/h^+^ pair separation, as well as to the synergistic effect of the high area of the open hollow ellipsoidal capsule’s structure of Ti-Fe_2_O_3_ and the increased donor density.

## Supplementary Information

ESM 1 (DOCX 572 kb)
