# Supplementary figures and images for: Eco-friendly and solar light-active Ti-Fe2O3 ellipsoidal capsules’ nanostructure for removal of herbicides and organic dyes
Source: Environ Sci Pollut Res Int. 2022 Oct 6;30(7):17765–75. doi: 10.1007/s11356-022-23119-0 (PMC9929020; doi:10.1007/s11356-022-23119-0)

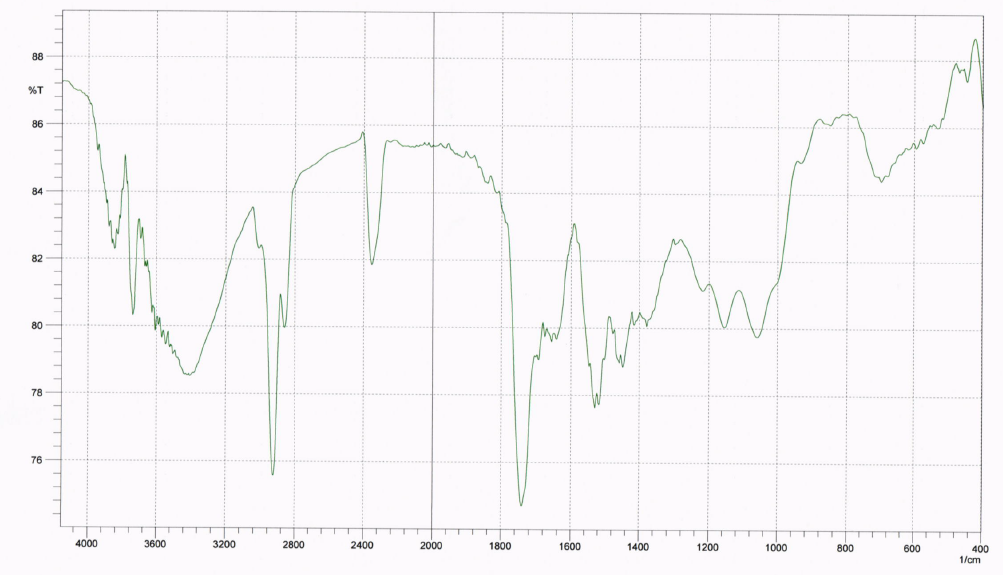


(a)

(b)


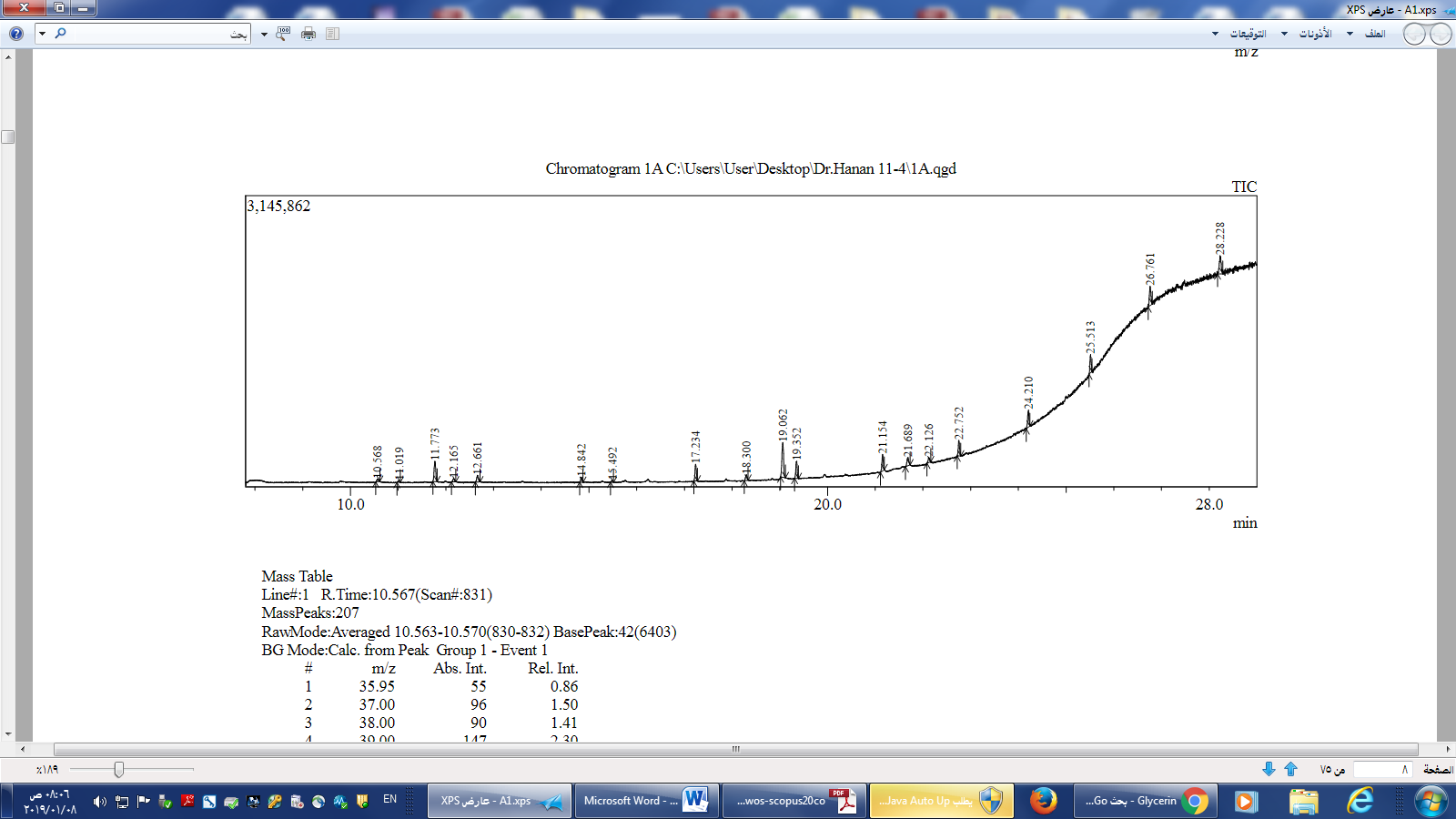


(c)

**Figure S1.** UV-vis DRS (a) FTIR (b) and chromatogram (c) of the solid FSE.

Supplement: Supplementary file 1 — (DOCX 572 kb) [file 11356_2022_23119_MOESM1_ESM.docx]
